# Common and personal target genes of the micronutrient vitamin D in primary immune cells from human peripheral blood

**DOI:** 10.1038/s41598-020-78288-0

**Published:** 2020-12-03

**Authors:** Andrea Hanel, Antonio Neme, Marjo Malinen, Emmi Hämäläinen, Henna-Riikka Malmberg, Stéphane Etheve, Tomi-Pekka Tuomainen, Jyrki K. Virtanen, Igor Bendik, Carsten Carlberg

**Affiliations:** 1 School of Medicine, Institute of Biomedicine, University of Eastern Finland, POB 1627, 70211 Kuopio, Finland; 2 Institute for Applied Mathematics, Merida Research Unit, National Autonomous University of Mexico, Sierra Papacal, Merida, Yucatan Mexico; 3 Department of Environmental and Biological Sciences, University of Eastern Finland, Joensuu, Finland; 4 DSM Nutritional Products Inc., R&D Solution Center, Kaiseraugst, Switzerland; 5 Institute of Public Health and Clinical Nutrition, University of Eastern Finland, Kuopio, Finland; 6 DSM Nutritional Products Inc., Human Nutrition and Health, Kaiseraugst, Switzerland

**Keywords:** Computational biology and bioinformatics, Genetics, Immunology, Molecular biology, Endocrinology

## Abstract

Vitamin D is essential for the function of the immune system. In this study, we treated peripheral blood mononuclear cells (PBMCs) of healthy adults with the biologically active form of vitamin D_3_, 1α,25-dihydroxyvitamin D_3_ (1,25(OH)_2_D_3_) using two different approaches: single repeats with PBMCs obtained from a cohort of 12 individuals and personalized analysis based on triplicates of five study participants. This identified 877 (cohort approach) and 3951 (personalized approach) genes that significantly (p < 0.05) changed their expression 24 h after 1,25(OH)_2_D_3_ stimulation. From these, 333 and 1232 were classified as supertargets, a third of which were identified as novel. Individuals differed largely in their vitamin D response not only by the magnitude of expression change but also by their personal selection of (super)target genes. Functional analysis of the target genes suggested the overarching role of vitamin D in the regulation of metabolism, proliferation and differentiation, but in particular in the control of functions mediated by the innate and adaptive immune system, such as responses to infectious diseases and chronic inflammatory disorders. In conclusion, immune cells are an important target of vitamin D and common genes may serve as biomarkers for personal responses to the micronutrient.

## Introduction

The micronutrient vitamin D_3_ is essential for calcium homeostasis and bone formation^1,2^ but also an important modulator of innate and adaptive immunity in their fight against infections^3^ and the prevention of autoimmune diseases^4^, respectively. Serum levels of 25-hydroxyvitamin D_3_ (25(OH)D_3_), which is the most stable vitamin D_3_ metabolite, serve as a biomarker of the vitamin D status of individuals^5^. A 25(OH)D serum level below 50 nM may lead to musculoskeletal disorders, such as rickets in children and osteomalacia and fractures in adults^6^. Moreover, vitamin D insufficiency is linked to a large set of immunologic disorders, such as multiple sclerosis^7^, inflammatory bowel disease^8^, rheumatoid arthritis^9^, type I diabetes^10^, or infections like tuberculosis^11^ as well as to cancers of colon, breast, prostate and blood^12^. Furthermore, individuals differ in their response to supplementation with vitamin D_3_, such as biochemical and physiological changes^13^, which led to the concept of the personal vitamin D response index^14^. The molecular details of this personal response to vitamin D are yet not fully understood but seem to be based on differential gene activity in individuals^15,16^.

Vitamin D_3_ acts a pre-hormone, since its biologically most active metabolite 1,25(OH)_2_D_3_ is the high affinity ligand to the transcription factor vitamin D receptor (VDR)^17^. VDR is expressed in most human tissues, in each of which it has a few hundred target genes^18,19^. Thus, the key molecular mechanisms of the actions of vitamin D in health and disease are changes in the transcriptome in its target tissues. Preceding to these transcriptome changes are effects of vitamin D on the epigenome of these tissues and cell types^20^, which is represented by covalent and structural modifications of chromatin^21^. The latter comprise histone modifications^22^, changes in chromatin accessibility^23^ as well as VDR binding to the genome^24^. One of the most relevant methods for investigating the epigenome is chromatin immunoprecipitation sequencing (ChIP-seq), which for VDR detected more than 23,000 sites along different human cell types^24^.

**Figure 1:**
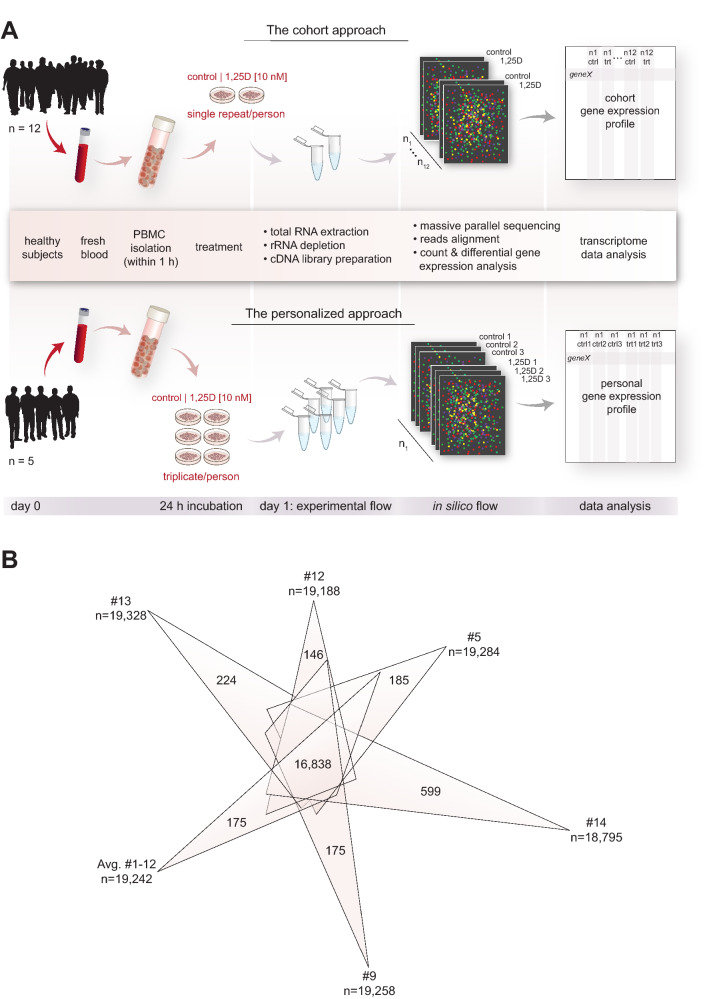
Studying gene expression in PBMCs. Design of the study (**A**). PBMCs of 12 healthy individuals (Table 1) were isolated and treated for 24 h with 1,25(OH)_2_D_3_ (1,25D) or solvent (0.1% EtOH) in single repeat (**top**). With PBMCs of five individuals (three of which already participated in the cohort approach study) 1,25(OH)_2_D_3_ and solvent treatment was performed in triplicate (**bottom**). RNA was extracted and RNA-seq analysis was performed for basal gene expression and 1,25(OH)_2_D_3_ stimulated expression. A Venn diagram was used, in order to display commonly and personally expressed genes in the average of the cohort approach and in the five individuals that were investigated in triplicate (**B**).

At present, the most comprehensive investigation of vitamin D signaling on the level of the transcriptome and epigenome was performed in the human monocytic leukemia cell line THP-1^25^. A straightforward approach to extend this model to easily accessible primary human cell types is the use of PBMCs, which are primarily composed of lymphocytes and monocytes^19^. In this study, we took advantage of that in context of the vitamin D intervention trial VitDHiD (NCT03537027), in which PBMCs of healthy adults were isolated for in vitro investigations. For a total of 14 participants of the study we used RNA sequencing (RNA-seq), in order to investigate changes in the transcriptome of PBMCs in response to a 24 h stimulation with 1,25(OH)_2_D_3_. We used two different study designs to detect significant changes in the epigenome (Fig. 1A): single repeats of 12 individuals formed the cohort approach, while triplicates of five participants described the personalized approach. The integration of the two approaches allowed the identification of a number of common vitamin D target genes as well as of many genes that are personal, *i.e.*, specific to a subset of the study participants. Thus, gene expression changes of cells from peripheral blood may serve as a biomarker for common as well as personal responses to vitamin D.

## Material and methods

### Sample collection

This report serves as a 1,25(OH)_2_D_3_ reference of the VitDHiD trial, the main goal of which was the investigation of the response of individuals to 25(OH)D_3_. Blood samples were collected after overnight (12 h) fasting from healthy individuals (age 21–54, body mass index (BMI) 21.4–25.6, Table 1) for serum and PBMC isolation. Serum 25(OH)D_3_ concentrations were measured using UPLC (1290 Infinity II LC System, Agilent) coupled with MS detection (API 4000 LC–MS/MS System, SCIEX). Individuals were selected based on their basal 25(OH)D_3_ levels, in order to represent a wide range (50 to 125 nM, Table 1). The study took place in May 2018, *i.e.*, after the end of Finnish winter. The ethics committee of the Northern Savo Hospital District had approved the study protocol (#515/2018). All participants gave written informed consent to participate in the study. All experiments were performed in accordance with relevant guidelines and regulations.

**Table 1:** Study participants.

# participant	Age	Gender	BMI	25(OH)D_3_ (nM)
1	21	F	22.6	125
2	21	F	21.7	65
3	33	M	22.7	57
4	23	F	23.9	86
5	27	M	23.0	77
6	23	F	22.2	78
7	21	M	23.5	112
8	21	F	24.5	95
9	41	M	23.2	80
10	21	M	21.4	54
11	36	F	22.4	50
12	24	M	24.1	86
13	26	M	25.6	61
14	54	M	25.1	118

Age, gender and BMI are indicated and 25(OH)D_3_ serum levels at the time of PBMC isolation are indicated. Single repeat PBMC stimulation and RNA-seq had been performed for participants #1–12, while triplicate assays were done with cells of participants #5, 9, 12, 13 and 14.

### PBMC isolation and culture

PBMCs were isolated within one hour after collection of 20 ml peripheral blood using Vacutainer CPT Cell Preparation Tubes with sodium citrate (Becton Dickinson) according to manufacturer’s instructions. After washing with phosphate-buffered saline the cells were either stored in liquid nitrogen until use or immediately grown in RPMI 1640 medium supplemented with 10% charcoal-depleted fetal calf serum, 2 mM L-glutamine, 0.1 mg/ml streptomycin and 100 U/ml penicillin and were exposed for 24 h to either 10 nM 1,25(OH)_2_D_3_ (Sigma-Aldrich) or solvent (0.1% EtOH) at 37 °C in a humidified 95% air/5% CO_2_ incubator.

### RNA-seq analysis

Total RNA was extracted using the High Pure RNA Isolation kit (Roche) following the manufacturer’s protocol. RNA quality was assessed on an Agilent Bioanalyzer and library preparation was performed after rRNA depletion applying kits and protocols from New England Biolabs. RNA-seq libraries were sequenced at 75 bp read length on a NextSeq 500 system (Illumina) using standard protocols at the Gene Core of the EMBL (Heidelberg, Germany). Fastq files of the raw data can be found at Gene Expression Omnibus (GEO, www.ncbi.nlm.nih.gov/geo) with accession number GSE156124. After quality control using *afterQC*^26^, RNA-seq analysis was conducted via high-quality reads through the application of *kallisto*^27^ with parameters –b 100 –single –I 180 –s 20. This software approximates abundance in a fast and efficient way via a pseudo-alignment stage to the reference genome (hg38). Genes were considered expressed when their normalized expression exceeded the value of 0.5. Differential gene expression was computed using *DESeq2*^28^ and *EdgeR*^29^, both of which implement a negative binomial test over the reads in the two conditions (treated/untreated), with standard parameters and a p-value cutoff of 0.05. By employing an empirical Bayes approach, these two tools are robust enough to detect differential expression in relatively low numbers of replicates, which describe the situation in this study. Based on previous differential gene expression analyses^30^, we defined those genes with a relative change in expression above 2 between conditions as supertargets.

### Epigenomic characterization of vitamin D target genes

Published VDR ChIP-seq data from human monocytic leukemia cells (THP-1^23,30^) and immortalized B cell clones of two HapMap individuals (GM10855 and GM10861^31^) that had been treated with 1,25(OH)_2_D_3_ or solvent (EtOH) were used. The IGV browser^32^ was applied to visualize VDR bearing enhancers up- and downstream of the genes’ transcription start sites. VDR binding sites were classified into “persistent” (present all times), “transient” (present only at a few time points) or “24 h only” (present only after a vitamin D stimulation for 24 h) based on time course data obtained in THP-1 cells^30^.

### Data analysis

Human genome nomenclature committee (HGNC) gene symbols of all datasets were brought up to date using the R package HGNC helper (version 0.8.1, https://CRAN.R-project.org/package=HGNChelper) and annotated with gene identifiers, description, genomic location and biotype by accessing the Ensembl database (release 100) via the R package BiomaRt (version 2.42.1^33^). Entrez Gene identifiers were added with the R package org.Hs.eg.db (version 3.10.0) and incomplete mappings of target genes manually retrieved from NCBI (www.ncbi.nlm.nih.gov/home/genes). Genes missing genomic position information or being mitochondrially encoded were removed. Functional analysis was performed using the Signaling Pathway Impact Analysis (SPIA) algorithm^34^ implemented in the R package SPIA (version 2.38.0). SPIA is a topology-aware pathway analysis method that considers interactions and dependencies between genes, *i.e.*, it outperforms methods which ignore pathway structures, such as over-representation analysis and gene set enrichment analysis, by an improved specificity and more relevant pathway ranking^35^. SPIA was performed with the setting nB = 2000 on Entrez ID-annotated vitamin D target genes. Current pathways of the Kyoto Encyclopedia of Genes and Genomes (KEGG) database (release 95.0) were downloaded through REST-style KEGG API (http://rest.kegg.jp/list/pathway/hsa) and the respective KGML (xml) files parsed by the SPIA function makeSPIAdata. Data were visualized using the R packages Complexheatmap (2.2.0^36^), GenomicRanges (1.38.0^37^) and ggbio (1.34.0^38^) and Venn diagrams were created via the application http://jvenn.toulouse.inra.fr/app.

## Results

### Basal gene expression in PBMCs

Freshly isolated PBMCs from in total 14 healthy adults (Table 1) were used in this study for in vitro stimulation with 1,25(OH)_2_D_3_. In a first approach, PBMCs of 12 individuals, who covered the wide range of 25(OH)D_3_ serum levels from of 50–125 nM, were treated for 24 h in a single repeat with 1,25(OH)_2_D_3_ or solvent (Fig. 1A, top). In a second approach, PBMCs of participants #5, #9 and #12, who showed mid-range 25(OH)D_3_ levels of 77–86 nM, and those of two further individuals, who displayed a low (#13, 61 nM) and high (#14, 118 nM) vitamin D status (Table 1), were selected and in vitro stimulation with 1,25(OH)_2_D_3_ and solvent was performed in three biological repeats (Fig. 1A, bottom). RNA-seq analysis was executed for the cohort approach (24 samples, Table S1) as well as for the personalized approach (30 samples, Table S2). From the total set of 32,978 genes the cohort approach indicated 15,244 commonly expressed genes as well as 10,194 genes that were not expressed in any of the 12 individuals (Fig. S1A). This implies that within the group of 12 persons 7540 genes (22.9% of all) are expressed in a somehow personalized fashion. For example, there are 63 to 239 genes that were expressed only in one of the participants as well as 18 to 177 genes that were not expressed only in one of the 12 study participants (Fig. S1A). This produced a range of 19,166 to 17,831 expressed genes in individuals #1–12.

The personalized approach resulted in a range of 19,328 to 18,795 expressed genes for the five selected persons (Fig. S1B). Together with the average of the cohort approach (19,242 genes), 16,838 commonly expressed genes were identified, while 11,281 genes were not found to be transcribed in PBMCs of any of the five individuals or the average of the cohort approach. Strikingly, 146 to 599 genes are personally expressed exclusively in the PBMCs of one of the five study participants or the average of the cohort approach (Fig. 1B), while 741, 657, 675 and 1282 genes are shared between exactly two, three, four or five groups, respectively. Thus, overall 4859 genes (14.7% of all) showed individual-specific expression.

In summary, in vitro stimulation of PBMCs using either single repeats of 12 individuals (cohort approach) or triplicates of five study participants (personalized approach) identified approximately 16,800 commonly expressed genes but also some 4800 genes expressed in a personal fashion.

### Vitamin D target genes in PBMCs: the cohort approach

**Figure 2:**
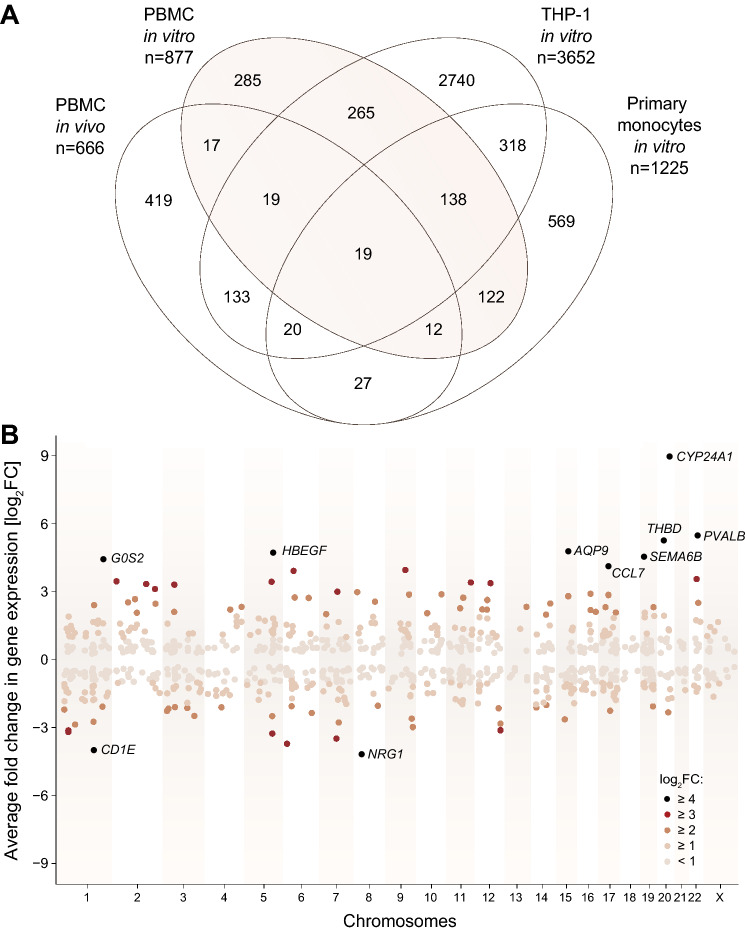
Genome-wide view on vitamin D target genes in PBMCs. Based on PBMCs isolated from a cohort of 12 individuals and treated in vitro in a single repeat with 1,25(OH)_2_D_3_ (Fig. 1A, top) differential gene expression after RNA-seq analysis identified 877 vitamin D target genes (Table S1). A Venn diagram was used for displaying the overlap of this set of vitamin D target genes with those identified from a cohort of five individuals, which had been exposed in vivo to a bolus of 2000 µg vitamin D_3_^16^, THP-1 cells, which had been stimulated in vitro with 1,25(OH)_2_D_3_^23^, and monocytes isolated from PBMCs of a cohort of 85 individuals, which also had been stimulated in vitro with 1,25(OH)_2_D_3_^39^ (**A**). A Manhattan plot displays the genome-wide distribution of the 877 vitamin D target genes and indicates their responsiveness (**B**). The 10 most responsive genes (FC > 16) are highlighted.

The 19,242 genes that represent the average gene expression profile of PBMCs derived from the cohort of 12 individuals were analyzed for significant (p < 0.05) regulation by 1,25(OH)_2_D_3_ compared to solvent control (Table S1). These 877 (4.6%) vitamin D target genes displayed an overlap with 67 genes, which had been described previously in PBMCs of a cohort of five individuals being exposed in vivo to a bolus of 2000 µg (80,000 IU) vitamin D_3_^16^, with 441 genes of the monocytic cell line THP-1, which had been stimulated in vitro with 1,25(OH)_2_D_3_^23^, and with 291 genes of monocytes isolated from PBMCs of a cohort of 85 individuals, which also had been stimulated in vitro with 1,25(OH)_2_D_3_^39^ (Fig. 2A). Thus, 592 of the 877 genes (67.5%) were already known to respond to vitamin D in PBMCs or monocytes, but the remaining 285 genes are novel vitamin D targets in the human hematopoietic system (Table S1).

Genome-wide, the 877 vitamin D target genes are equally distributed (Fig. 2B), 402 (45.8%) of which were up-regulated and 475 (54.2%) down-regulated. Most of these genes (837) are protein coding, but there are also 39 pseudogenes and one non-coding RNA (ncRNA) gene (Table S1). A subset of 333 of these 877 genes showed a fold change (FC) > 2 in expression and were referred to as supertargets. From these, 159 (47.7%) were up-regulated and 174 (52.3%) down-regulated, 298 were protein coding, 33 pseudogenes and one a ncRNA gene (Table S1). However, there was a larger number of genes showing an extreme up-regulation (> 16-fold) than comparable down-regulation. The up-regulated genes *CYP24A1* (cytochrome P450 family 24 subfamily A member 1), *G0S2* (G0/G1 switch 2), *HBEGF* (heparin binding EGF like growth factor), *SEMA6B* (semaphoring 6B) and *THBD* (thrombomodulin) were already known from studies in THP-1 cells^23^. In contrast, the up-regulated genes *AQP9* (aquaporin 9), *CCL7* (C–C motif chemokine ligand 7) and *PVALB* (parvalbumin) and the down-regulated genes *CD1E* (CD1e molecule) and *NRG1* (neuregulin 1) are novel vitamin D targets.

Calculating the average of a number of individual single treatments, such as described here for the cohort of 12 individuals, hides interindividual differences. More detailed inspection of the 10 above described, most responsive vitamin D target genes indicated that there were rather large differences between the individual concerning the magnitude in the responsive of their genes (Fig. S2). Moreover, in PBMCs of a few individuals some of the vitamin D target genes were even not expressed. For example, the well-known vitamin D target gene *CYP24A1* was in average 506-fold up-regulated, which was based on 11 individuals ranging in their *CYP24A1* expression changes between 105- and 2020-fold, while in one individual the gene was not expressed in non-stimulated cells (*i.e.*, no FC could be calculated) (Table S1).

In order to visualize changes of the epigenome in the genomic regions of the above mentioned vitamin D supertarget genes via vitamin D-triggered VDR binding to enhancer regions, we used previously published VDR ChIP-seq data from THP-1 monocytes^23,30^ and from two immortalized B cell clones^31^. The representative examples of the *G0S2* locus (Fig. S3A), the *HBEGF* gene (Fig. S3B) and the *AQP9* gene (Fig. S3C) indicated that there are prominent vitamin D-sensitive VDR sites 15 kB upstream, 4.2 kb downstream and 8.7 kb downstream of the gene’s transcription start sites, respectively. For each locus the most prominent VDR binding sites were selected based on their classification in THP-1 cells^30^. Interestingly, the VDR bearing enhancer of the *G0S2* gene is most likely also regulating the neighboring vitamin D supertargets *LAMB3* (laminin subunit beta 3) and *HSD11B1* (hydroxysteroid 11-beta dehydrogenase 1).

Taken together, based on average gene expression in PBMCs of 12 individuals 877 vitamin D target genes were identified, one third of which had not been described previously in PBMCs or monocytes. Moreover, 333 of these genes were vitamin D supertargets and showed rather large interindividual differences in their expression and responsiveness.

### Vitamin D target genes in PBMCs: the personalized approach

**Figure 3:**
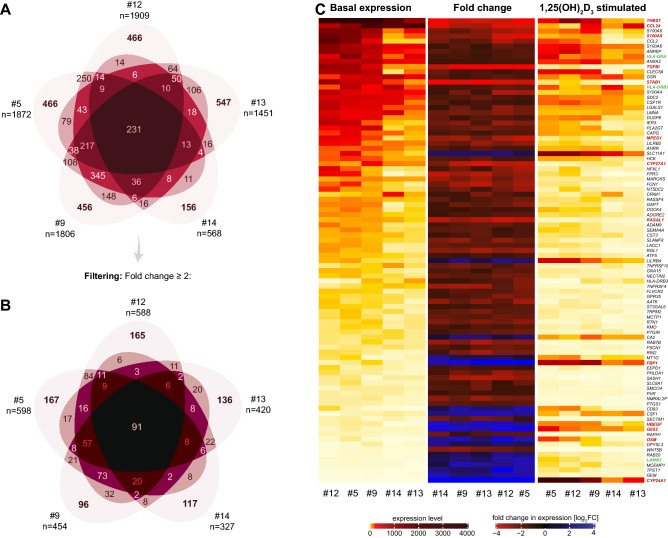
Common and personal vitamin D target genes in PBMCs. Based on PBMCs isolated from five individuals and treated in vitro in triplicate with 1,25(OH)_2_D_3_ (Fig. 1A, bottom) differential gene expression after RNA-seq analysis identified 568 to 1909 vitamin D target genes per individual, 327 to 598 of which are supertargets (Table S1). Venn diagrams were used for displaying the overlap of the respective five sets of vitamin D target genes (**A**) and supertargets (**B**). A heatmap was used, in order to compare the basal expression (**left**), FC (**center**) and 1,25(OH)_2_D_3_-induced expression (**right**) of the 91 vitamin D supertarget genes (**C**). Sorting was by their average basal expression. Genes that change their expression more than eightfold (Fig. 4A) are highlighted in red, while those that are the drivers of common pathways (Fig. 4B) are in green.

The datasets obtained from PBMCs that were treated in triplicate (personalized approach) were analyzed separately for each of the five individuals concerning significant (p < 0.05) regulation of gene expression by 1,25(OH)_2_D_3_ (Table S2). This resulted in 1909, 1872, 1806, 1451 and 568 vitamin D target genes for individuals #12, #5, #9, #13 and #14, respectively, *i.e.*, 3.0–9.9% of the total number of their expressed genes seemed to be under control of vitamin D. Interestingly, only 231 vitamin D target genes were common to all five persons, while 547 (#13), 466 (both #12 and #5), 456 (#9) and 156 (#14) genes, *i.e.*, in total 2091 genes, turned out to be exclusively personal targets (Fig. 3A). Moreover, 812 genes were shared only by two individuals, 532 by three and 285 by four, *i.e.*, together the five individuals claimed 3951 vitamin D targets, the large majority (94.2%) of which were at least in part personal. From these 3951 genes 351 were pseudogenes and 21 ncRNA genes (Table S2).

Filtering the 3951 vitamin D target genes for supertargets resulted in 598, 588, 454, 420 and 327 genes for individuals #5, #12, #9, #13 and #14, respectively, from which 167 (#5), 165 (#12), 136 (#13), 117 (#14) and 96 (#9) were exclusive personal supertargets (Fig. 3B). These in total 681 exclusive personal vitamin D supertargets were contrasted with 91 genes that were common to all five individuals. Furthermore, 100 genes were shared by four persons, 131 by three and 229 by two. Thus, in total 1232 vitamin D supertarget genes were identified, most of which (92.6%) were at least in part personal. The personal supertargets showed pseudogene and ncRNA rates 30.2 and 1.8%, respectively, while the 91 common supertargets comprised of only one pseudogene (Table S2).

From the 3951 target genes, 255 showed a personal response to 1,25(OH)_2_D_3_ because they were expressed only in a subset of the individuals. They had either a very low basal expression, such as *COL4A2* (collagen type IV alpha 2 chain, Fig. S4A), or they were down-regulated below a threshold value, such as *IL6* (interleukin 6, Fig. S4B). However, the majority of the personal vitamin D target genes displayed individual-specific regulation, such as *INSR* (insulin receptor, Fig. S4C) and *BCL2* (BCL2 apoptosis regulator, Fig. S4D). Nevertheless, the epigenetic profile of the representative personal target genes *COL4A2* (Fig. S5A), *INSR* (Fig. S5B) and *BCL2* (Fig. S5C) indicated at least one enhancer showing 1,25(OH)_2_D_3_-dependent VDR binding in both monocytes and B cells per gene locus. Thus, primary common and personal vitamin D target genes seem to follow the same mechanisms of regulation via at least one VDR binding enhancer located within the genomic locus of the gene.

The 91 common vitamin D supertarget genes differed in their magnitude of expression between the five tested individuals. A heatmap, in which the genes were sorted by their average basal expression, illustrates that the interindividual differences are based on both a difference in the basal gene expression levels as well as in the expression after stimulation with 1,25(OH)_2_D_3_ (Fig. 3C). In consequence, only a minority of the 91 genes showed a common strength in their response to 1,25(OH)_2_D_3_. Nevertheless, the average change in expression of the eight down-regulated genes *CCL24* (C–C motif chemokine ligand 24*)*, *CYP27A1* (cytochrome P450 family 27 subfamily A member 1), *MPEG1* (macrophage expressed 1), *RASAL1* (RAS protein activator like 1), *S100A9* (S100 calcium binding protein A9), *STAB1* (stabilin 1), *TGFBI* (transforming growth factor beta induced) and *THBS1* (thrombospondin 1) or the five up-regulated genes *CYP24A1*, *FBP1* (fructose-bisphosphatase 1), *G0S2*, *HBEGF* and *OSM* (oncostatin M) was found to be more than eightfold. Interestingly, the genes *CCL24*, *MPEG1*, *RASAL1*, *STAB1* and *TGFBI* have not yet been described in the context of vitamin D, *i.e.*, they are novel vitamin D targets.

The personalized approach confirmed 674 (76.9%) of the 877 vitamin D target genes, which had been identified in the cohort approach (Fig. S6A). Similarly, 249 (74.8%) of the 333 vitamin D supertargets of the cohort approach were found by the personalized approach (Fig. S6B). However, only 51 of the 91 common supertargets (56.0%) of the personalized approach had already been identified by the cohort approach (Fig. S6C).

In summary, analyzing PBMCs of the same individual in triplicate (personalized approach) resulted in four of five cases in a clearly higher number of vitamin D target genes and supertargets than the single analysis of a cohort of individuals (cohort approach). Most of the genes, which were additionally identified in the personalized approach, are personal vitamin D targets, while the number of common targets (231) and supertargets (91) is low. The VDR binding pattern suggests identical mechanisms in the regulation of personal and common supertarget genes. However, the expression profile of both type of supertargets is divergent between the tested individuals.

### Functional analysis of vitamin D target genes

**Figure 4:**
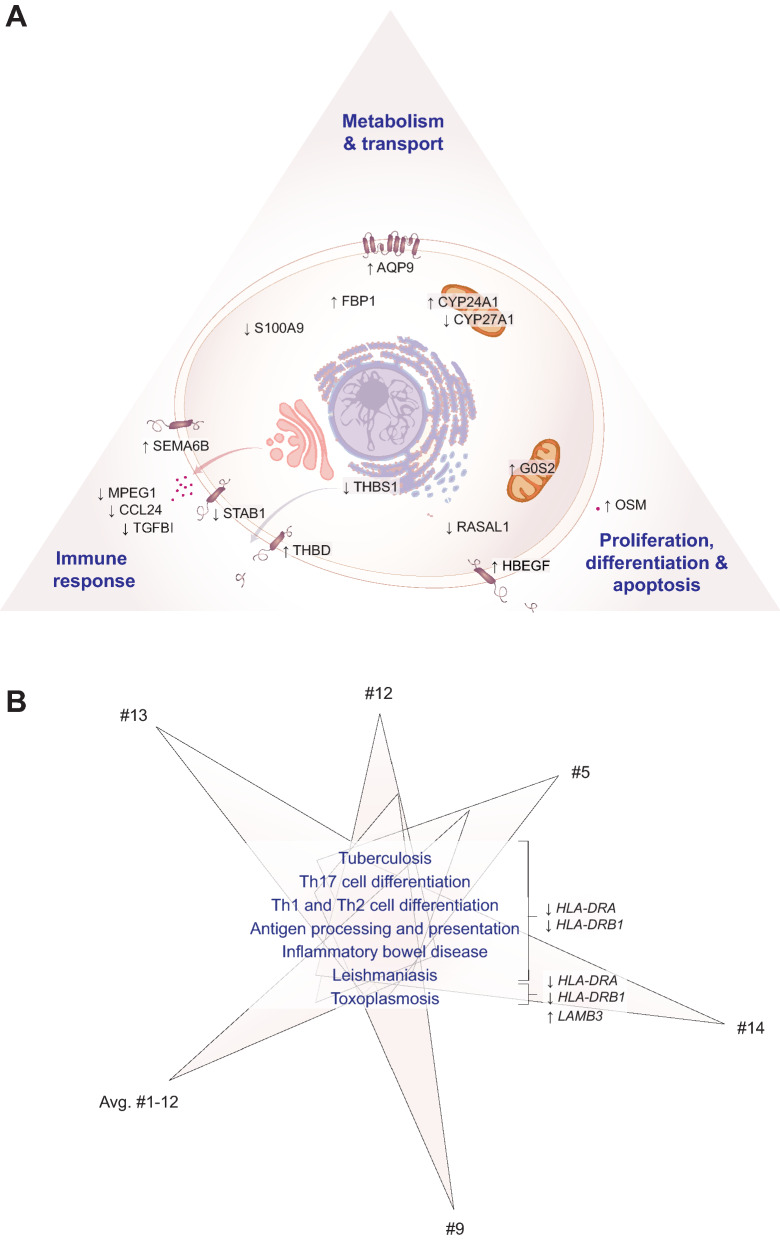
Functional profile of main vitamin D target genes. Schematic picture of a cell indicating the main location of the proteins encoded by the most responsive (cohort: FC > 16, personalized: FC > 8), common vitamin D target genes (**A**). Information based on the integrative database GeneCards (www.genecards.org) and literature allowed the classification of the encoded proteins into the functional groups “immune response”, “metabolism & transport” and “proliferation, differentiation & apoptosis”. SPIA pathway analysis was performed using vitamin D target genes identified based on the cohort approach (Fig. 2) and the personalized approach (Fig. 3A). A Venn diagram indicates seven common pathways that are primarily based on the indicated supertarget genes (**B**). The orientation of vertical arrows indicates up- and down-regulated genes.

From the 13 common vitamin D supertargets that showed in the personalized approach a FC > 8 in their expression (Table S2) *CCL24*, *CYP24A1*, *FBP1*, *G0S2*, *HBEGF*, *OSM* and *TGFBI* were already identified by the cohort approach (Table S1) as genes that prominently change their expression. In contrast, from the 10 most responsive genes of the cohort approach, *CCL7*, *CD1E*, *NRG1* and *PVALB* were not found as prominent targets in the personalized approach. Therefore, we focused on a functional analysis on the 13 common vitamin D supertargets of the personalized approach together with the genes *AQP9*, *SEMA6B* and *THBD* highlighted by the cohort approach. Based on information provided by the integrative database GeneCards and inspection of the literature^40–42^, the predominant cellular location of the proteins encoded by these 16 genes was determined (Fig. 4A). Each five of the proteins are acting in the plasma membrane or are secreted, *i.e.*, they belong to those proteins, by which the cell takes contact with micro-environment. Furthermore, each three of the proteins encoded by the vitamin D target genes are located in the cytoplasm or in mitochondria. Based on cellular locations, identified protein domains and known functions, the proteins AQP9, CYP24A1, CYP27A1, FBP1 and S100A9 are involved in cellular metabolism and transport, the proteins G0S2, HBEGF, OSM and RASAL1 act on the control of cellular proliferation, differentiation and apoptosis, while the proteins CCL24, MPEG1, SEMA6B, STAB1, TGFBI, THBD and THBS1 function in the context of immunity. Thus, like in most other vitamin D target tissues, the function of vitamin D target genes can be subdivided into the control of metabolism, cellular growth and immune responses.

An alternative, computational approach of functional analysis was conducted via the SPIA algorithm by testing which pathways within PBMCs were significantly impacted by stimulation with 1,25(OH)_2_D_3_. The advantage of this method is its capacity to integrate high-throughput data, *i.e*., the complete list of differentially expressed genes, including their interactions and dependencies between genes, in a more global context. SPIA uses the information of the KEGG database, which is a compilation of manually verified maps for biological pathways^43^. We performed SPIA analysis with vitamin D target genes being identified both via the cohort and the personalized approach, for which Entrez IDs were available (Table S3). When applying the stringent Bonferroni adjusted p-value threshold of 0.001, all six datasets have seven pathways in common, which were “Th (T helper) 1, Th2 and Th17 cell differentiation”, “antigen processing and presentation”, “inflammatory bowel disease”, “tuberculosis”, “leishmaniasis” and “toxoplasmosis” (Fig. 4B). Moreover, based on the 51 common supertargets of the cohort and personalized approach (Fig. S6C) the down-regulated genes *HLA-DRA* and *HLA-DRB1* were found to be prominently involved in all seven highlighted pathways, while the up-regulated gene *LAMB3* (Fig. S3A) played an additional role in toxoplasmosis.

Taken together, the manual inspection of the 16 most responsive common genes as well as computational pathway analysis highlights vitamin D as a regulator of innate and adaptive immunity controlling chronic inflammation disorders as well as infectious diseases.

## Discussion

Vitamin D is a direct regulator of gene expression and transcriptome-wide studies of the effects of the micronutrient are master examples in the field of nutrigenomics^44^. In this line, the main aim of this study was the description of changes in the transcriptome of PBMCs, which had been freshly isolated from healthy individuals, in response to an in vitro stimulation with 1,25(OH)_2_D_3_. A traditional approach of investigating PBMCs of a cohort of individuals in a single repeat was compared with testing a limited number of persons in triplicate, *i.e.*, in a personalized way. Strikingly, although the PBMCs were isolated and stimulated in an identical fashion, rather different numbers of 877 and 3951 genes were found to change statistically significantly (p < 0.05) their expression in the cohort and the personalized approach, respectively. In contrast, the number of expressed genes showed a rather narrow range of 17,831 to 19,328 genes for all tested participants. Thus, the experimental design of a study is critical for the number of target genes that can be identified.

Numerous studies in different vitamin D target tissues, such as in colon cancer cells^45^ or monocytic leukemia cells^46,47^, already reported high counts of target genes, when statistical significance (*i.e.,* p-value threshold) was applied as the exclusive criterium. This suggested the need of further filtering procedures like a threshold for relative changes in gene expression, such as 2-fold^30^, or a focus on vitamin D target genes with highest basal expression^48^. Furthermore, the comparison of different cell types or donors would highlight the most important genes, *i.e*., the most elegant approach would be single cell analysis^49^ rather than testing a bulk of PBMCs.

In this study, we applied the twofold gene expression change threshold and focused on 333 and 1232 vitamin D supertargets in the cohort and the personalized approach, respectively. After 24 h in vitro stimulation with 1,25(OH)_2_D_3_ more genes are down-regulated than up-regulated. This tendency was also observed previously in other cell culture systems^12,18^. Vitamin D time course studies^23^ indicate that most of the down-regulated genes are secondary targets, *i.e.*, that their regulation is not the result of VDR binding to their enhancer region but rather caused by transcription factors, such as BCL6^50^, and chromatin modifiers like the demethylase KDM6B^51^ that are encoded by primary vitamin D target genes.

Many of the vitamin D target genes identified in this study had already been described in previous reports. However, one third of the genes are novel targets and provide a new perspective on the function of vitamin D in lymphocytes and monocytes. Interestingly, key novel vitamin D target genes, such as *CCL24*, *CD1E*, *MPEG1*, *NRG1*, *RASAL1*, *STAB1* and *TGFBI*, are prominently down-regulated by stimulation with 1,25(OH)_2_D_3_. Together with already previously known vitamin D target genes, such as *CYP24A1*, *FBP1*, *G0S2, THBD* and *HBEGF*, these genes are in the shortlist of serving as biomarkers for the vitamin D response of human peripheral white blood cells. For example, the in vivo response of the genes *THBD*^13,52^, *G0S2*^53^ and *FBP1*^54^ within PBMCs of vitamin D_3_ supplemented individuals already had been used for their segregation into high, mid and low responders.

The personalized approach has the additional option to focus on 231 common vitamin D targets and 91 vitamin D supertargets. This allows to summarize vitamin D responses on the level of the transcriptome and their functional consequences that can be expected also by any other individual. However, common vitamin D targets and supertargets represent the surprisingly low number of 5.8 or 7.4%, respectively, of all identified target genes. Accordingly, the vast number of the here reported vitamin D target genes in PBMCs have a personal component. However, it should be noted that personal target genes bear a higher risk of being false positives, in contrast to common genes which were independently “confirmed” by a number of participants. Thus, setting a more stringent significance threshold than the standard cut-off (*i.e*., p-value < 0.05) as well adjusting for multiple hypothesis testing would mitigate this uncertainty.

Some 250 low expressed genes, such as *COL4A2*, are personal because they show an individual-specific expression. Similarly, higher gene expression threshold settings during data analysis would suppress this apparent transcriptional noise, but in this study, we adjusted the threshold to the low basal expression of the very specific vitamin D target gene *CYP24A1*. The latter encodes for an enzyme controling the levels of 25(OH)D_3_ and 1,25(OH)_2_D_3_, *i.e.*, the expression threshold was chosen low enough for detecting *CYP24A1* as an expressed target gene. A master example of a personal vitamin D target is the *CCL7* gene that in the cohort approach is expressed in 7 out of 12 individuals, in which its induction varies between 2.3 and 31-fold. Similarly, in the personalized approach the *CCL7* gene is expressed in 3 of 5 individuals, but is a vitamin D target in only one person. The personal nature of expression and regulation of the *CCL7* gene may be the main reason, why it had not been detected earlier as a vitamin D target.

Other prominent vitamin D target genes, such as *CAMP* (cathelicidin antimicrobial peptide), *IL6* or *CD14*, show in primary cells of healthy individuals rather low basal expression, since they are only expressed at higher levels in case of infections or tissue injuries. Accordingly, some genes are personal depending on the health and lifestyle situation of the individual. This implies that individuals may respond differently to a nutrient or drug depending on their current life challenges. This individual responses can be detected in personalized gene expression approaches and are often lost in cohort approaches that are based on averages. Thus, the number of vitamin D target is some 4-times higher in the personalized approach, because a large number of personal target genes can be identified.

The majority of the personal vitamin D targets seem to have an individual-specific regulation. This may be the key molecular explanation for the phenomenon of the personal vitamin D response index^14^. However, there is no indication of different mechanisms of gene regulation between individuals but more likely the genomic regions of some VDR binding sites may either not be accessible or carry polymorphisms in the VDR binding motif. Thus, epigenetic and genetic variations may explain the personal nature of vitamin D target genes^55^.

The functional analysis of the most responsive common vitamin D target genes in PBMCs indicate the regulation of metabolism, cell growth and innate and adaptive immunity as the prominent role of the micronutrient in leukocytes demonstrating that the pleiotropy of vitamin D signaling has an evolutionary origin^56^. Computational pathway analysis additionally highlighted the genes *HLA-DRA* and *HLA-DRB1*, which encode for components of the major histocompatibility complex (MHC) II, as common drivers of the functional profile of vitamin D in PBMCs. Interestingly, components of MHC I, *HLA-A* and *HLA-C*, which locate at the same cluster in human chromosome 6^57^, had been identified as vitamin D targets within in vivo stimulated human PBMCs^16^. MHC II proteins are only expressed in antigen-presenting cells, such as dendritic cells, macrophages and B cells, while MHC I is found on all nucleated cells of the body. Since (i) the SPIA pathway algorithm considers genes encoding for proteins occurring in a beginning of a pathway (*i.e*., receptors) to have a greater impact on its functionality than genes occurring somewhere downstream^34^ and (ii) *HLA* genes belong to the most studied genes of the human genome, it is not surprising that KEGG-based pathway analysis highlighted *HLA-DRA* and *HLA-DRB1* as the most prominent vitamin D targets within the 51 common supertargets. Nevertheless, this finding emphasizes the key role of vitamin D in regulating innate and adaptive immune system.

Finally, this study focused on the actions of 1,25(OH)_2_D_3_ being generated by the actions of CYP2R1 and CYP27B1 and activating VDR. However, there is growing evidence that CYP11A1 is responsible for alternative metabolism of vitamin D and its precursor 7-dehydrocholesterol ^58,59^ generating 20(OH)D_3_ and 20,23(OH)_2_D_3_^60–64^. These hydroxyderivatives were shown to act as reverse agonists of the nuclear receptors RORα and RORγ^65,66^ in the skin. Since immune cells express CYP11A1^67^, other vitamin D metabolites may as well contribute to the immunomodulatory actions of vitamin D. Thus, future analysis should also take alternative vitamin D metabolites into account.

In conclusion, we compared a cohort approach for the identification of vitamin D target genes in PBMCs with a personalized approach and found clear advantages for the latter. This suggests that repeated analysis of individuals is more powerful in detecting novel target genes than the single investigation of cohorts. Moreover, in this way the concept of personal responses to vitamin D can be understood on a molecular level. Some of the key vitamin D target genes may be used as biomarkers for the segregation of individuals in high, mid or low responders.

## Supplementary information

Supplementary Information 1.

Supplementary Information 2.

Supplementary Information 3.

Supplementary Information 4.

## Abbreviations

1,25(OH)_2_D_3_ or 1,25D: 1α,25-Dihydroxyvitamin D_3_
25(OH)D_3_: 25-Hydroxyvitamin D_3_
AQP9: Aquaporin 9
BCL2: BCL2 apoptosis regulator
BMI: Body mass index
CAMP: Cathelicidin antimicrobial peptide
CCL: C-C Motif chemokine ligand
CD: Cluster of differentiation
ChIP-seq: Chromatin immunoprecipitation sequencing
COL4A2: Collagen type IV alpha 2 chain
CYP: Cytochrome P450
FBP1: Fructose-bisphosphatase 1
FC: Fold change
FE: Fold enrichment
G0S2: G0/G1 switch 2
HBEGF: Heparin binding EGF like growth factor
HGNC: Human genome nomenclature committee
HLA: Human leukocyte antigen
HSD11B1: Hydroxysteroid 11-beta dehydrogenase 1
IGV: Integrative Genomics Viewer
IL6: Interleukin 6
INSR: Insulin receptor
KEGG: Kyoto Encyclopedia of Genes and Genomes
LAMB3: Laminin subunit beta 3
MHC: Major histocompatibility complex
MPEG1: Macrophage expressed 1
ncRNA: Non-coding RNA
NRG1: Neuregulin 1
OSM: Oncostatin M
PBMC: Peripheral blood mononuclear cell
PVALB: Parvalbumin
RAB20: RAB20, member RAS oncogene family
RASAL1: RAS protein activator like 1
RNA-seq: RNA sequencing
S100A9: S100 calcium binding protein A9
SEMA6B: Semaphorin 6B
SPIA: Signaling Pathway Impact Analysis
STAB1: Stabilin 1
TGFBI: Transforming growth factor beta induced
Th: T helper
THBD: Thrombomodulin
THBS1: Thrombospondin 1
VDR: Vitamin D receptor

## References

[1] van de Peppel J, van Leeuwen JP (2014). Vitamin D and gene networks in human osteoblasts. Front. Physiol..

[2] Carmeliet G, Dermauw V, Bouillon R (2015). Vitamin D signaling in calcium and bone homeostasis: a delicate balance. Best Pract. Res. Clin. Endocrinol. Metab..

[3] Chun RF, Liu PT, Modlin RL, Adams JS, Hewison M (2014). Impact of vitamin D on immune function: lessons learned from genome-wide analysis. Front. Physiol..

[4] Lu M, McComish BJ, Burdon KP, Taylor BV, Körner H (2019). The association between vitamin D and multiple sclerosis risk: 1,25(OH)_2_D_3_ induces super-enhancers bound by VDR. Front. Immunol..

[5] Hollis BW (2005). Circulating 25-hydroxyvitamin D levels indicative of vitamin D sufficiency: implications for establishing a new effective dietary intake recommendation for vitamin D. J. Nutr..

[6] Bouillon R (2008). Vitamin D and human health: lessons from vitamin D receptor null mice. Endocr. Rev..

[7] Sintzel MB, Rametta M, Reder AT (2018). Vitamin D and multiple sclerosis: a comprehensive review. Neurol. Ther..

[8] Fletcher J, Cooper SC, Ghosh S, Hewison M (2019). The role of vitamin D in Inflammatory bowel disease: mechanism to management. Nutrients.

[9] Jeffery LE, Raza K, Hewison M (2016). Vitamin D in rheumatoid arthritis-towards clinical application. Nat. Rev. Rheumatol..

[10] Infante M (2019). Influence of vitamin D on islet autoimmunity and beta-cell function in type 1 diabetes. Nutrients.

[11] Huang SJ (2017). Vitamin D deficiency and the risk of tuberculosis: a meta-analysis. Drug Des. Dev. Ther..

[12] Carlberg C, Munoz A (2020). An update on vitamin D signaling and cancer. Semin. Cancer Biol..

[13] Carlberg C (2013). Primary vitamin D target genes allow a categorization of possible benefits of vitamin D_3_ supplementation. PLoS ONE.

[14] Carlberg C, Haq A (2018). The concept of the personal vitamin D response index. J. Steroid Biochem. Mol. Biol..

[15] Seuter S (2017). Molecular evaluation of vitamin D responsiveness of healthy young adults. J. Steroid Biochem. Mol. Biol..

[16] Neme A (2019). *In vivo* transcriptome changes of human white blood cells in response to vitamin D. J. Steroid Biochem. Mol. Biol..

[17] Haussler MR (2008). Vitamin D receptor: molecular signaling and actions of nutritional ligands in disease prevention. Nutr. Rev..

[18] Campbell MJ (2014). Vitamin D and the RNA transcriptome: more than mRNA regulation. Front. Physiol..

[19] Carlberg C (2018). Vitamin D genomics: from in vitro to in vivo. Front. Endocrinol..

[20] Fetahu IS, Hobaus J, Kallay E (2014). Vitamin D and the epigenome. Front. Physiol..

[21] Rivera CM, Ren B (2013). Mapping human epigenomes. Cell.

[22] Nurminen V, Neme A, Seuter S, Carlberg C (2018). The impact of the vitamin D-modulated epigenome on VDR target gene regulation. Biochim. Biophys. Acta.

[23] Seuter S, Neme A, Carlberg C (2016). Epigenome-wide effects of vitamin D and their impact on the transcriptome of human monocytes involve CTCF. Nucl. Acids Res..

[24] Tuoresmäki P, Väisänen S, Neme A, Heikkinen S, Carlberg C (2014). Patterns of genome-wide VDR locations. PLoS ONE.

[25] Carlberg C (2019). Vitamin D signaling in the context of innate immunity: focus on human monocytes. Front. Immunol..

[26] Chen S (2017). AfterQC: automatic filtering, trimming, error removing and quality control for fastq data. BMC Bioinform..

[27] Bray NL, Pimentel H, Melsted P, Pachter L (2016). Near-optimal probabilistic RNA-seq quantification. Nat. Biotechnol..

[28] Love MI, Huber W, Anders S (2014). Moderated estimation of fold change and dispersion for RNA-seq data with DESeq2. Genome Biol..

[29] Robinson MD, McCarthy DJ, Smyth GK (2010). edgeR: a Bioconductor package for differential expression analysis of digital gene expression data. Bioinformatics.

[30] Neme A, Seuter S, Carlberg C (1860). Selective regulation of biological processes by vitamin D based on the spatio-temporal cistrome of its receptor. Biochim. Biophys. Acta.

[31] Ramagopalan SV (2010). A ChIP-seq defined genome-wide map of vitamin D receptor binding: associations with disease and evolution. Genome Res..

[32] Thorvaldsdottir H, Robinson JT, Mesirov JP (2013). Integrative Genomics Viewer (IGV): high-performance genomics data visualization and exploration. Brief. Bioinform..

[33] Durinck S, Spellman PT, Birney E, Huber W (2009). Mapping identifiers for the integration of genomic datasets with the R/Bioconductor package biomaRt. Nat. Protoc..

[34] Tarca AL (2009). A novel signaling pathway impact analysis. Bioinformatics.

[35] Vanhaelen Q, Aliper AM, Zhavoronkov A (2017). A comparative review of computational methods for pathway perturbation analysis: dynamical and topological perspectives. Mol. BioSyst..

[36] Gu Z, Eils R, Schlesner M (2016). Complex heatmaps reveal patterns and correlations in multidimensional genomic data. Bioinformatics.

[37] Lawrence M (2013). Software for computing and annotating genomic ranges. PLoS Comput. Biol..

[38] Yin T, Cook D, Lawrence M (2012). ggbio: an R package for extending the grammar of graphics for genomic data. Genome Biol..

[39] Kariuki SN (2016). Mapping variation in cellular and transcriptional response to 1,25-Dihydroxyvitamin D_3_ in peripheral blood mononuclear cells. PLoS ONE.

[40] Ma CY (2012). Monocytic thrombomodulin triggers LPS- and gram-negative bacteria-induced inflammatory response. J. Immunol..

[41] Shabani F, Farasat A, Mahdavi M, Gheibi N (2018). Calprotectin (S100A8/S100A9): a key protein between inflammation and cancer. Inflamm. Res..

[42] Ramadori G (2019). S100A9 extends lifespan in insulin deficiency. Nat. Commun..

[43] Kanehisa M, Furumichi M, Tanabe M, Sato Y, Morishima K (2017). KEGG: new perspectives on genomes, pathways, diseases and drugs. Nucl. Acids Res..

[44] Carlberg C (2019). Nutrigenomics of vitamin D. Nutrients.

[45] Palmer HG (2003). Genetic signatures of differentiation induced by 1α,25-dihydroxyvitamin D_3_ in human colon cancer cells. Cancer Res..

[46] Verway M (2013). Vitamin D induces interleukin-1beta expression: paracrine macrophage epithelial signaling controls *M. tuberculosis* infection. PLoS Pathog..

[47] Nurminen V, Seuter S, Carlberg C (2019). Primary vitamin D target genes of human monocytes. Front. Physiol..

[48] Hanel A, Malmberg HR, Carlberg C (2020). Genome-wide effects of chromatin on vitamin D signaling. J. Mol. Endocrinol..

[49] Olsen KS, Skeie G, Lund E (2015). Whole-blood gene expression profiles in large-scale epidemiological studies: what do they tell?. Curr. Nutr. Rep..

[50] Nurminen V (1849). The transcriptional regulator BCL6 participates in the secondary gene regulatory response to vitamin D. Biochim. Biophys. Acta.

[51] Pereira F (2011). KDM6B/JMJD3 histone demethylase is induced by vitamin D and modulates its effects in colon cancer cells. Hum. Mol. Genet..

[52] Ryynänen J (2014). Changes in vitamin D target gene expression in adipose tissue monitor the vitamin D response of human individuals. Mol. Nutr. Food Res..

[53] Saksa N (2015). Dissecting high from low responders in a vitamin D_3_ intervention study. J. Steroid Biochem. Mol. Biol..

[54] Vukic M (2015). Relevance of vitamin D receptor target genes for monitoring the vitamin D responsiveness of primary human cells. PLoS ONE.

[55] Hanel A, Carlberg C (2020). Skin color and vitamin D: an update. Exp. Dermatol..

[56] Hanel A, Carlberg C (2020). Vitamin D and evolution: pharmacologic implications. Biochem. Pharmacol..

[57] Trowsdale J, Knight JC (2013). Major histocompatibility complex genomics and human disease. Annu. Rev. Genom. Hum. Genet..

[58] Slominski A (2004). A novel pathway for sequential transformation of 7-dehydrocholesterol and expression of the P450scc system in mammalian skin. Eur. J. Biochem..

[59] Slominski AT (2012). Cytochrome P450scc-dependent metabolism of 7-dehydrocholesterol in placenta and epidermal keratinocytes. Int. J. Biochem. Cell Biol..

[60] Slominski A (2005). The cytochrome P450scc system opens an alternate pathway of vitamin D3 metabolism. FEBS J..

[61] Slominski AT (2015). Novel activities of CYP11A1 and their potential physiological significance. J. Steroid Biochem. Mol. Biol..

[62] Slominski AT (2014). In vivo production of novel vitamin D2 hydroxy-derivatives by human placentas, epidermal keratinocytes, Caco-2 colon cells and the adrenal gland. Mol. Cell Endocrinol..

[63] Slominski AT (2012). In vivo evidence for a novel pathway of vitamin D_3_ metabolism initiated by P450scc and modified by CYP27B1. FASEB J..

[64] Slominski AT (2015). Detection of novel CYP11A1-derived secosteroids in the human epidermis and serum and pig adrenal gland. Sci. Rep..

[65] Slominski AT (2014). RORalpha and RORgamma are expressed in human skin and serve as receptors for endogenously produced noncalcemic 20-hydroxy- and 20,23-dihydroxyvitamin D. FASEB J..

[66] Slominski AT (2017). Endogenously produced nonclassical vitamin D hydroxy-metabolites act as "biased" agonists on VDR and inverse agonists on RORα and RORγ. J. Steroid Biochem. Mol. Biol..

[67] Jia Y (2013). Steroidogenic enzyme Cyp11a1 regulates Type 2 CD8+ T cell skewing in allergic lung disease. Proc. Natl. Acad. Sci. USA.

