# Supplementary material for: Common and personal target genes of the micronutrient vitamin D in primary immune cells from human peripheral blood
Source: Sci Rep. 2020 Dec 3;10:21051. doi: 10.1038/s41598-020-78288-0 (PMC7713372; doi:10.1038/s41598-020-78288-0)
Supplement: Supplementary file 1 — Supplementary Information 1. [file 41598_2020_78288_MOESM1_ESM.pdf]

SUPPLEMENTARY MATERIAL

Common and personal target genes of the micronutrient vitamin D in primary immune cells from human peripheral blood

Andrea Hanel[1], Antonio Neme[2], Marjo Malinen[3], Emmi Hämäläinen[1], Henna-Riikka Malmberg[1], Stéphane Etheve[4], Tomi-Pekka Tuomainen[5], Jyrki K. Virtanen[5], Igor Bendik[6] and Carsten Carlberg[1,#]

[1]School of Medicine, Institute of Biomedicine, University of Eastern Finland, Kuopio, Finland

[2]Institute for Applied Mathematics, Merida Research Unit, National Autonomous University of Mexico, Sierra Papacal Merida Yucatan, Mexico

[3]Department of Environmental and Biological Sciences, University of Eastern Finland, Joensuu, Finland

[4]DSM Nutritional Products Inc., R&D Solution Center, Kaiseraugst, Switzerland

[5]Institute of Public Health and Clinical Nutrition, University of Eastern Finland, Kuopio, Finland

[6]DSM Nutritional Products Inc., Human Nutrition and Health, Kaiseraugst, Switzerland

Contents

Table S1: The vitamin D-triggered transcriptome of PBMCs (cohort approach).
Table S2: The vitamin D-triggered transcriptome of PBMCs (personalized approach).
Table S3: Significantly impacted pathways by vitamin D.
Fig. S1: Basal gene expression in PBMCs.
Fig. S2: Top vitamin D target genes found by the cohort approach.
Fig. S3: VDR bearing enhancers close to common vitamin D target genes.
Fig. S4: Profile of personal vitamin D target genes.
Fig. S5: VDR bearing enhancers close to personal vitamin D supertargets.
Fig. S6: Comparing vitamin D target genes.

SUPPLEMENTARY TABLES

Table S1: The vitamin D-triggered transcriptome of PBMCs (cohort approach). PBMCs of individuals #1-12 (**Table 1**) were isolated and treated in single repeat for 24 h with 10 nM 1,25(OH)$_2$D$_3$ (1,25D) or solvent (0.1% EtOH) (**Fig. 1A, top**). RNA was extracted and subjected to RNA-seq analysis. In total, 19,242 genes showed an average fold enrichment (FE) > 0.5 and were considered expressed (black), while the remaining 13,736 genes were not reproducibly expressed (grey). FC between 1,25(OH)$_2$D$_3$- and solvent-treated samples were calculated and based on all 12 individuals 877 expressed genes showed significant ($p < 0.05$) responses to 1,25(OH)$_2$D$_3$ (shaded grey), 333 of which are supertargets with absolute log2FC > 1. The most right-handed columns indicate, which genes were identified as vitamin D targets in PBMCs of a cohort of five individuals being exposed *in vivo* to a bolus of 2000 µg vitamin D$_3$[16], of THP-1 cells, which had been stimulated *in vitro* with 1,25(OH)$_2$D$_3$[23], and of monocytes isolated from PBMCs of a cohort of 88 individuals, which also had been stimulated *in vitro* with 1,25(OH)$_2$D$_3$[40].

Table S2: The vitamin D-triggered transcriptome of PBMCs (personalized approach). PBMCs of individuals #5, #9, #12, #13 and #14 (**Table 1**) were isolated and treated for 24 h with 1,25(OH)$_2$D$_3$ (1,25D) or solvent (0.1% EtOH) in triplicate (**Fig. 1A, bottom**). RNA was extracted and subjected to RNA-seq analysis. Genes showing FE > 0.5 and were considered expressed (black), while the remaining genes were not reproducibly expressed (grey). FC between 1,25(OH)$_2$D$_3$- and solvent-treated samples were calculated and individual-specific numbers of genes showed significant ($p < 0.05$) responses to 1,25(OH)$_2$D$_3$ (shaded green). The most right-handed columns indicate, which genes were identified as vitamin D targets or supertargets in the cohort approach (**Table S1**).

Table S3: Significantly impacted pathways by vitamin D. Functional analysis was performed using SPIA on Entrez-annotated vitamin D target genes of each individual of the

personalized approach as well the targets of the cohort approach, *i.e.*, on 1843, 1795, 1734, 1396, 540 and 850 targets of individuals #12, #5, #9, #13, #14 and #avg 1-12, respectively. Significant pathways (pGFWER < 0.001) after the stringent Bonferroni correction (FWER) are highlighted in blue. pGFWER, Bonferroni-adjusted global p-values.

SUPPLEMENTARY FIGURES

Fig. S1: Basal gene expression in PBMCs. RNA-seq analysis indicated the number of genes expressed in PBMCs of individuals #1-12 (single repeats, **A**) as well as of individuals #5, #9, #12, #13 and #14 (triplicates, **B**). Common and personally expressed (black dots) and non-expressed (grey dots) genes are indicated. The right border indicates the total number of expressed genes.

Fig. S2: Top vitamin D target genes found by the cohort approach. Boxplots are used, in order to display the interindividual differences in the inducibility of the 10 most responsive vitamin D target genes within PBMCs from 12 individuals (**Fig. 2**). Solid lines within the boxes indicate medians, while dashed lines mark the mean. The numbers on the right corner indicate in how many of the 12 individuals the respective genes are expressed.

Fig. S3: VDR bearing enhancers close to common vitamin D target genes. The Integrative Genomics Viewer (IGV)[33] we used in order to display VDR ChIP-seq data from THP-1 monocytes [23,31] and from two immortalized B cell clones[32] at the loci of the vitamin D supertargets *G0S2* (**A**), *HBEGF* (**B**) and *AQP9* (**C**) identified by the cohort approach. The VDR bearing enhancers around the *G0S2* gene most likely regulate in addition the neighboring vitamin D target genes *LAMB3* and *HSD11B1*. VDR-bound enhancers are shaded in grey, TSS regions of vitamin D target genes in red, and binding sites are classified as “persistent” (P), “transient” (T) and “24 h only” (24) based on a time course study in THP-1 cells [31].

Fig. S4: Profile of personal vitamin D target genes. The relative expression of the personal vitamin D target genes *COL4A2* (**A**), *IL6* (**B**), *INSR* (**C**) and *BCL2* (**D**) is displayed for individuals #5, #9, #12, #13 and #14. Bars represent the means of three independent biological replicates, error bars the standard deviations and asterisks the significance levels (** $p < 0.01$;

*** $p < 0.001$; **** $p < 0.0001$). Personal vitamin D supertarget genes (FC > 2) are highlighted in red.

Fig. S5: VDR bearing enhancers close to personal vitamin D supertargets. The Integrative Genomics Viewer (IGV)[33] we used to display VDR ChIP-seq data from THP-1 monocytes[23,31] and from two immortalized B cell clones [32] at the loci of the personal (1 of 5) supertarget *COL4A2* (**A**), the personal (2 of 5) supertarget *INSR* (**B**) and the personal (1 of 5) supertarget *BCL2* (**C**) identified by the personalized approach. The VDR bearing enhancers around the *COL4A2* gene most likely regulate in addition the neighboring common supertarget *RAB20*. VDR-bound enhancers are shaded in grey, TSS regions of vitamin D target genes in red, and binding sites are classified as “persistent” (P), “transient” (T) and “24 h only” (24) based on a time course study in THP-1 cells [31].

Fig. S6: Comparing vitamin D target genes. Venn diagrams were used, in order to compare the number of vitamin D target genes (**A**), supertargets (**B**) and common supertargets (**C**) identified in PBMCs using the cohort and the personalized approach.

Fig. S1

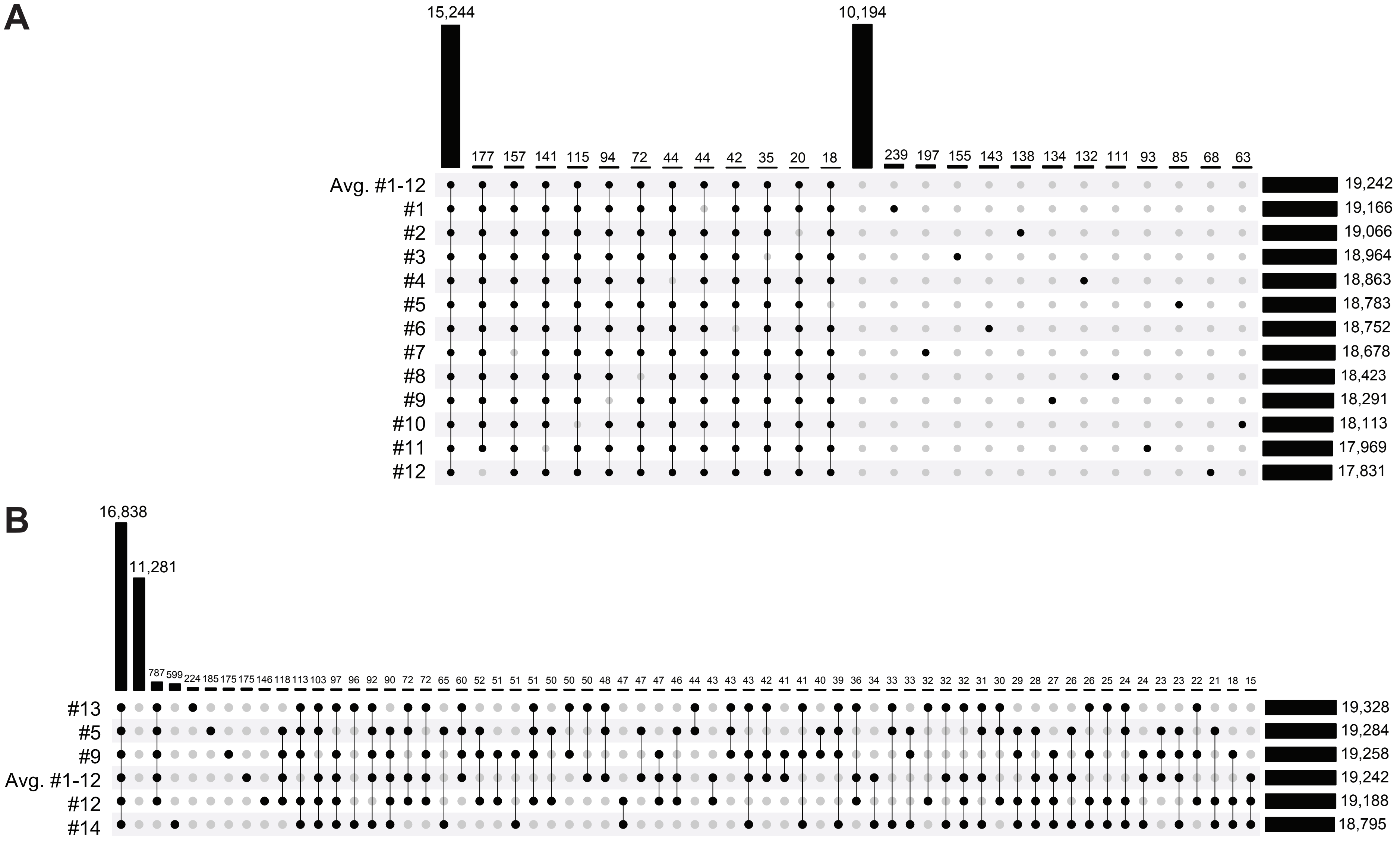

A
15,244
10,194
Avg. #1-12
#1
#2
#3
#4
#5
#6
#7
#8
#9
#10
#11
#12
19,242
19,166
19,066
18,964
18,863
18,783
18,752
18,678
18,423
18,291
18,113
17,969
17,831
B
16,838
11,281
#13
#5
#9
Avg. #1-12
#12
#14
19,328
19,284
19,258
19,242
19,188
18,795

Fig. S2

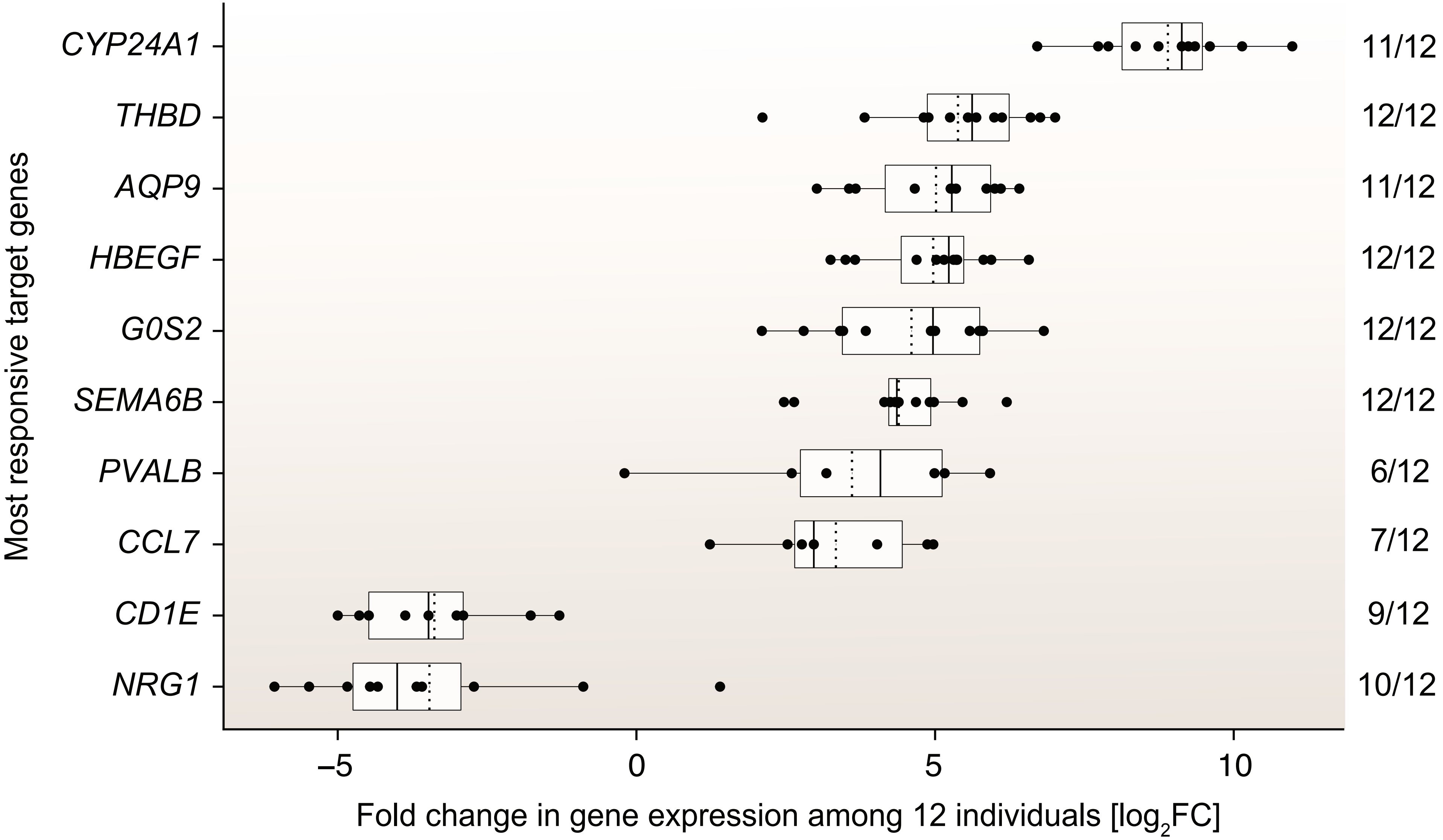

Most responsive target genes
CYP24A1
THBD
AQP9
HBEGF
G0S2
SEMA6B
PVALB
CCL7
CD1E
NRG1
11/12
12/12
11/12
12/12
12/12
12/12
6/12
7/12
9/12
10/12
–5
0
5
10
Fold change in gene expression among 12 individuals [log2FC]

Fig. S3

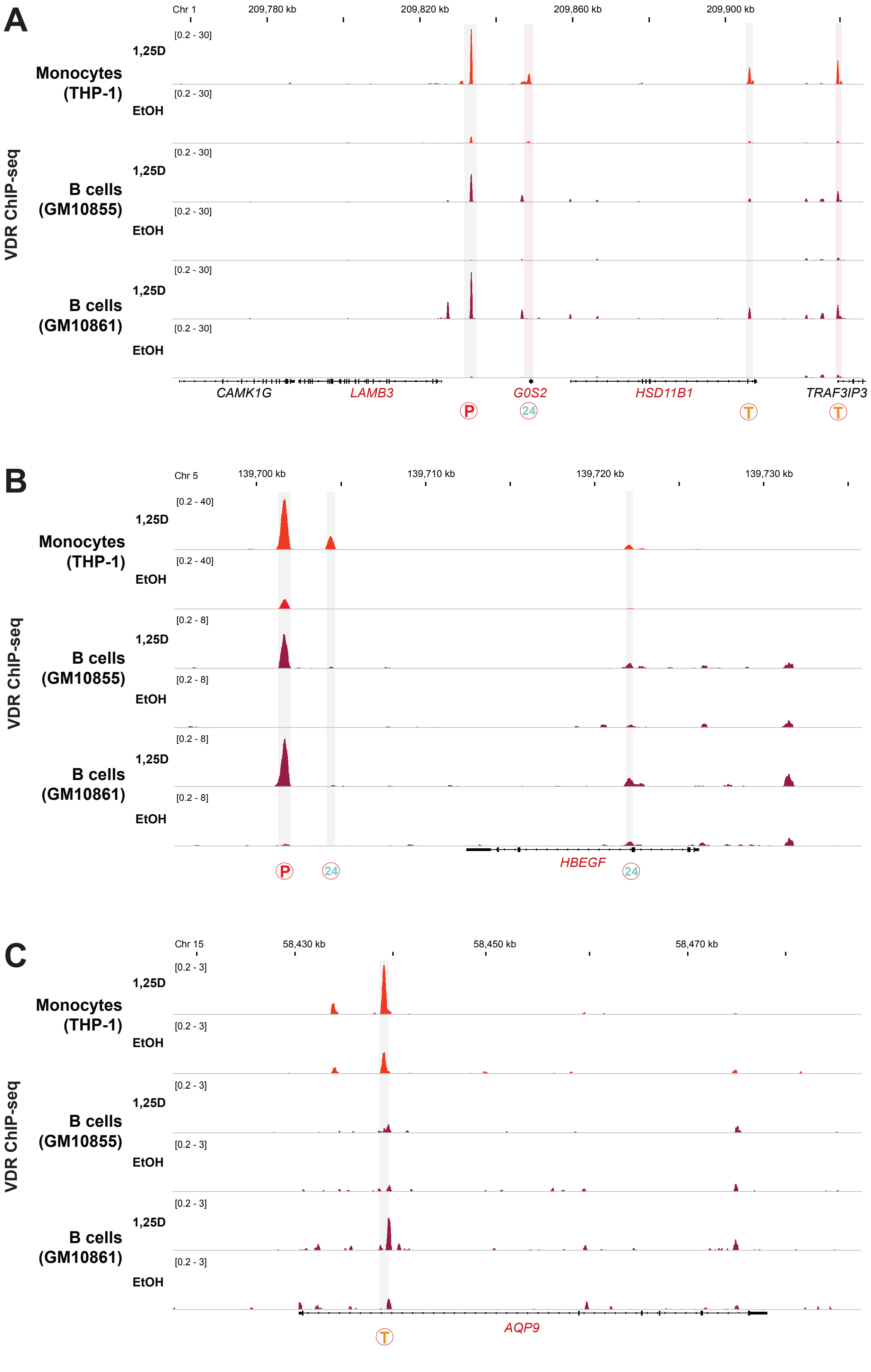

A
Chr 1
209,780 kb
209,820 kb
209,860 kb
209,900 kb
VDR ChIP-seq
Monocytes (THP-1)
B cells (GM10855)
B cells (GM10861)
1,25D
EtOH
[0.2 - 30]
CAMK1G
LAMB3
G0S2
HSD11B1
TRAF3IP3
P
24
T
B
Chr 5
139,700 kb
139,710 kb
139,720 kb
139,730 kb
[0.2 - 40]
[0.2 - 8]
HBEGF
C
Chr 15
58,430 kb
58,450 kb
58,470 kb
[0.2 - 3]
AQP9

Fig. S4

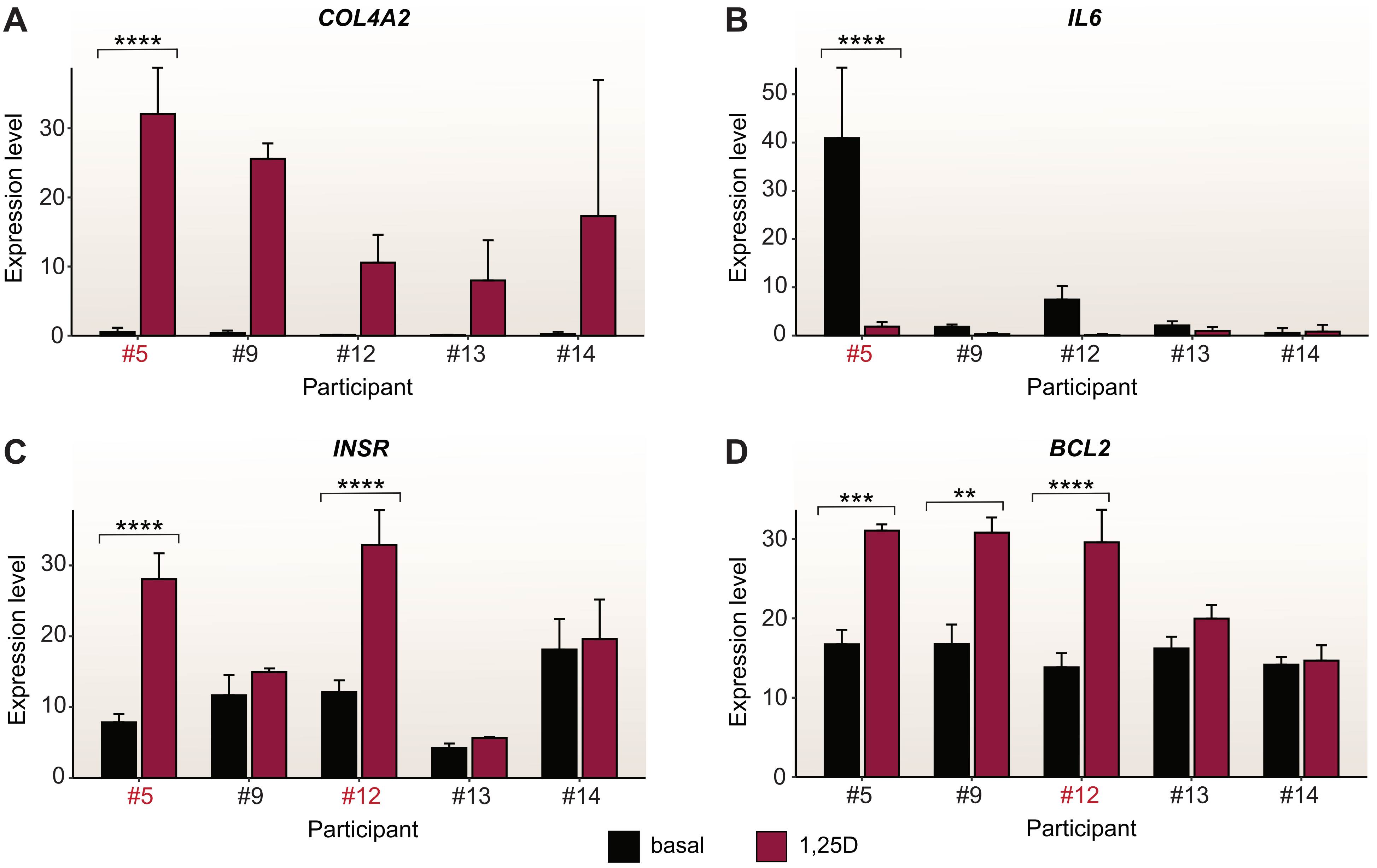
A
COL4A2
****
Expression level
0
10
20
30
#5
#9
#12
#13
#14
Participant
B
IL6
****
Expression level
0
10
20
30
40
50
#5
#9
#12
#13
#14
Participant
C
INSR
****
****
Expression level
0
10
20
30
#5
#9
#12
#13
#14
Participant
D
BCL2
***
**
****
Expression level
0
10
20
30
#5
#9
#12
#13
#14
Participant
basal
1,25D

Fig. S5

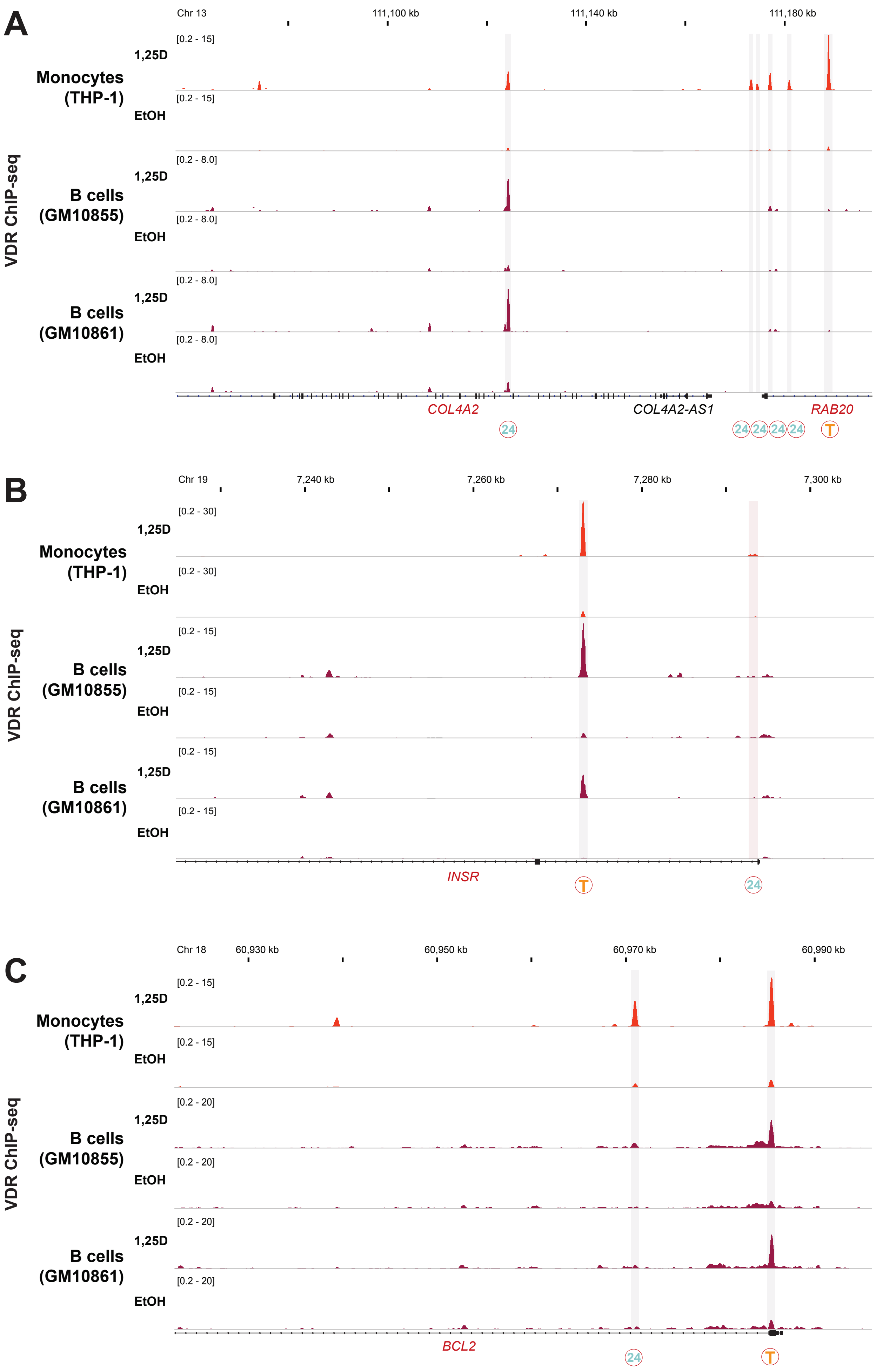
A
Chr 13
111,100 kb
111,140 kb
111,180 kb
VDR ChIP-seq
Monocytes (THP-1)
B cells (GM10855)
B cells (GM10861)
1,25D
EtOH
[0.2 - 15]
[0.2 - 8.0]
COL4A2
COL4A2-AS1
RAB20
24
T
B
Chr 19
7,240 kb
7,260 kb
7,280 kb
7,300 kb
[0.2 - 30]
[0.2 - 15]
INSR
C
Chr 18
60,930 kb
60,950 kb
60,970 kb
60,990 kb
[0.2 - 20]
BCL2

Fig. S6

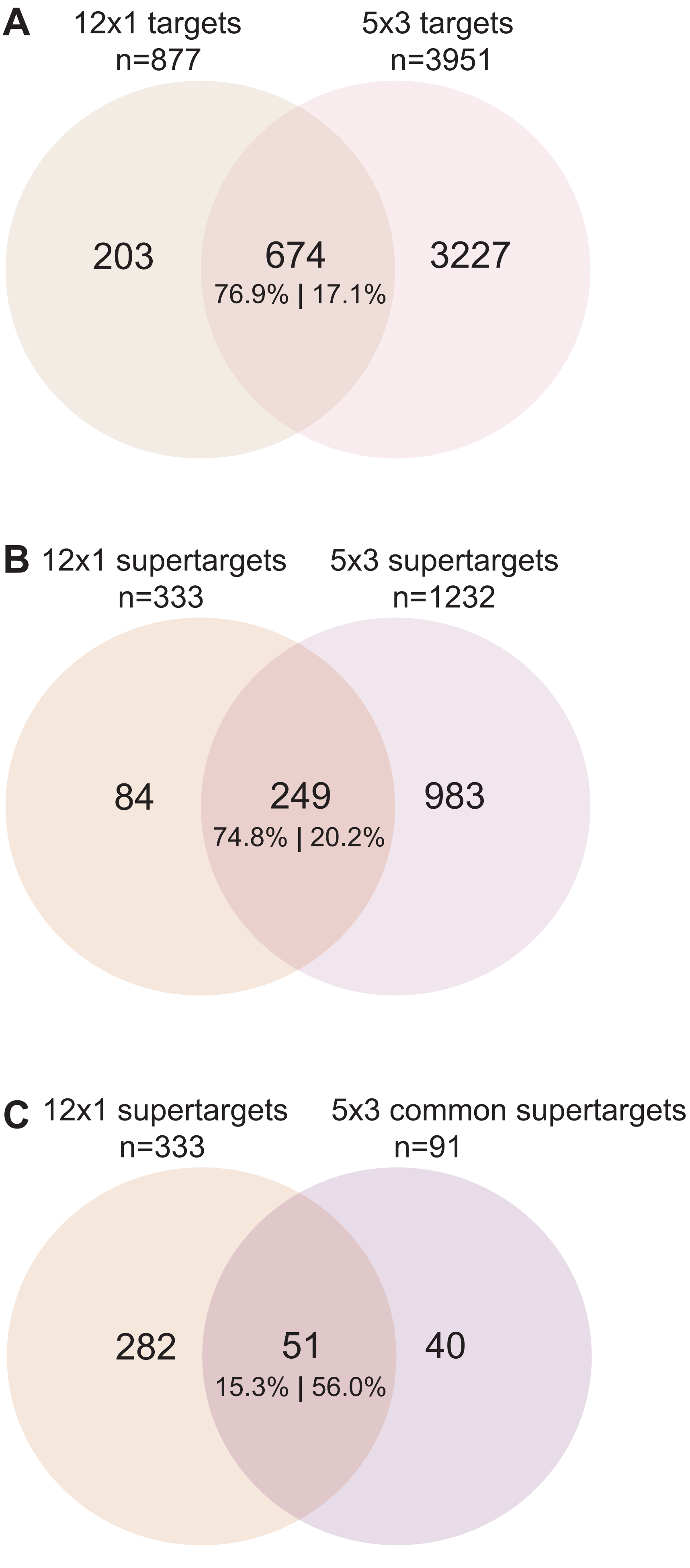
A
12x1 targets
n=877
5x3 targets
n=3951
203
674
76.9% | 17.1%
3227
B
12x1 supertargets
n=333
5x3 supertargets
n=1232
84
249
74.8% | 20.2%
983
C
12x1 supertargets
n=333
5x3 common supertargets
n=91
282
51
15.3% | 56.0%
40
